# A Case Report of Exacerbation of Leg Ulcers Associated with Acute High-dose Acetylsalicylic Acid in a Patient with Klinefelter Syndrome

**DOI:** 10.7759/cureus.6449

**Published:** 2019-12-23

**Authors:** Lena Arizanovic, Snezana Spasic, Cedo Miljevic, Mihajlo B Spasic, Milan Nikolic

**Affiliations:** 1 Biochemistry, University of Belgrade - Faculty of Chemistry, Belgrade, SRB; 2 Chemistry, Institute of Chemistry, Technology and Metallurgy, University of Belgrade, Belgrade, SRB; 3 Psychiatry, Institute of Mental Health, University of Belgrade - Faculty of Medicine, Belgrade, SRB; 4 Physiology, Institute for Biological Research "Sinisa Stankovic", University of Belgrade, Belgrade, SRB

**Keywords:** acetylsalicylic acid, testosterone, klinefelter syndrome, leg ulcers

## Abstract

Klinefelter syndrome (KS) is the most frequent type of congenital sex-chromosomal disorder caused by at least one extra X chromosome and commonly treated with lifetime testosterone therapy. Ulcerative lesions on lower extremities may occur as a complication of KS. The pathogenesis of ulcers in KS patients has not been clarified on a molecular level. Here we present a case of leg ulcers exacerbation associated with the administration of a high dose of acetylsalicylic acid in a 63-year-old KS patient with karyotype 47,XXY undergoing testosterone replacement therapy for the last 20 years. The appearance of the ulcer on the patient's leg occurred during one week of high oral acetylsalicylic acid intake (1.2 g daily). The patient was advised to return to his standard daily dose of 0.1 g of acetylsalicylic acid and significant improvement of his leg ulcer was observed after two weeks. We hypothesize that testosterone-mediated nitric oxide balance in KS patient is perturbed under the condition of acute high-dose acetylsalicylic acid administration. We propose that small standard doses of approximately 0.1 g/day of acetylsalicylic acid have no apparent effect on nitric oxide status, whereas higher doses may cause dysregulation of nitric oxide production and/or utilization, creating conditions which may cause the appearance of leg ulcers in the KS patients.

## Introduction

Klinefelter syndrome (KS) is the most frequent type of congenital sex-chromosomal disorder affecting approximately 1:660 newborn males, and it is a rather common cause of infertility, hypogonadism and learning disability [1]. Caused by at least one extra X chromosome, KS is presented with the genotype 47,XXY or variant forms 48,XXXY, 48,XXYY, 49,XXXXY and mosaic XY/XXY. It has been reported that KS patients have about 50% of normal testosterone levels. In addition, obesity, diabetes mellitus type 2, metabolic syndrome and a higher risk for cardiovascular disease are frequently observed as clinical complications of KS [1].

The occurrence of leg ulcers as a symptom of KS (6‒13%) appears to be related to occurrence of a single pathophysiological condition or a combination of several conditions such as chronic venous insufficiency, arterial dysplasia in lower extremities, platelet hyperaggregability, factor V Leiden mutation, and decrease in fibrinolysis due to elevated levels of plasminogen activator inhibitor-1 (PAI-1). However, the underlying molecular mechanism is not completely identified nor understood [2].

Due to the lack of reliable evidence, there is no consensus regarding the effect of oral intake of acetylsalicylic acid on venous leg ulcers [3]. We hereby report the case of a 63-year-old KS patient with 47,XXY genotype on a supplemental testosterone therapy who took 1.2 g of acetylsalicylic acid per day for a week. During this period of high-dose acetylsalicylic acid intake, the ulcer on the left leg of the KS patient appeared and gradually worsened. Significant healing of the ulcer was observed two weeks after he decreased the dose of acetylsalicylic acid (0.1 g daily), without any changes in the patient's standard therapy.

## Case presentation

The patient is a male born in 1956, diagnosed with KS at the age of 16. Regardless of a fact that KS is commonly associated with learning disabilities, the patient in question earned a Bachelor's degree in Economics at the age of 26. From 1982 to 2000 he was supplemented with human chorionic gonadotropin (Pregnyl® 5000 I.E., Organon, Austria), which was a recommendation of late professor and an expert in the management of KS treatment, Bruno Lunenfeld. In 2000, he becomes unresponsive to that therapy and began supplementation with testosterone-undecanoate (Nebido®, 1000 mg/4 mL solution for i.m. injections; Bayer, Germany) four times yearly (approximately every three months). The additional medications that he uses regularly are angiotensin-converting enzyme inhibitor Enalapril (Zdravlje Actavis, Serbia) and acetylsalicylic acid in a daily dose of 0.1 g/day as standard prescribed therapy for the management of high arterial blood pressure. Compiled from personal health record in the past 10 years, the patient had an average serum value for testosterone of 3.87 ± 1.14 ng/mL before injection of testosterone.

**Figure 1:**
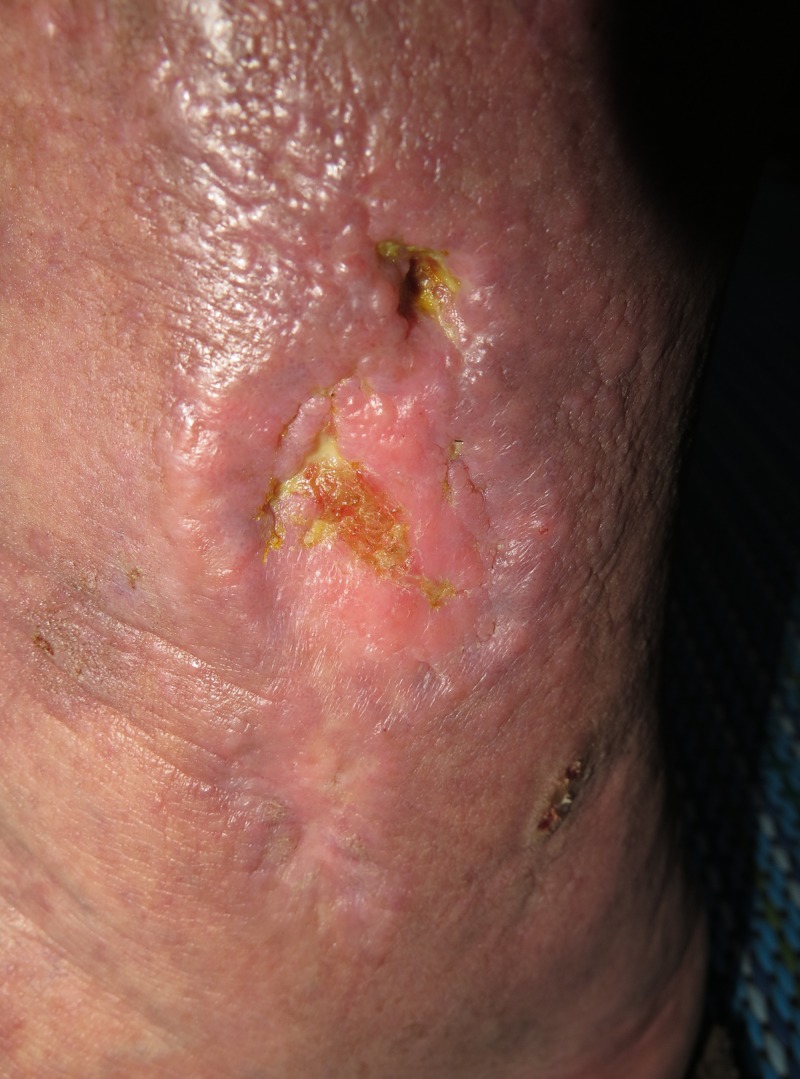
The appearance of left leg ulcer at presentation

In the past, the patient has experienced several episodes of leg ulceration often associated with an open bleeding wound that occurred simultaneously with the consumption of a high dose of acetylsalicylic acid. These problems started in mid-2014 after the patient voluntarily began taking increased doses of 400 mg of acetylsalicylic acid per day at times when he felt sick. One of the culminations happened on September 2018 when he took acetylsalicylic acid tablets (Aspirin® Plus C: 400 mg of acetylsalicylic acid and 240 mg of vitamin C; Bayer, Germany) three times a day, without prior consultation with his assigned physician. After a few days, an ulcer on his left leg re-appeared and worsened (Figure 1). In October 2018, the patient contacted us seeking advice. From the completed health/lifestyle assessment questionnaires, we found out that the patient is a non-smoker with regular eating habits, and with no other chronic condition except hypertension. His self-reported height and weight were 190 cm and 112 kg, respectively. The patient got a recommendation to return to the previous regimen of only 0.1 g of acetylsalicylic acid per day. It was observed that, upon following given recommendation, the ulcer on the patient's leg began to gradually decrease.

Clinical laboratory test results (from November and December 2018) are presented below, with normal/reference value or range given in brackets.

Laboratory evaluations showed a normal complete blood count, and blood group ABO & RhD B-. There were no abnormalities in the prothrombin time, partial thromboplastin time or plasminogen level. Fasting serum glucose was normal at 5.2 mM (3.9‒5.4 mM), as well as hemoglobin A1c level at 5.4% (normally less than 6%), indicating glucose homeostasis. The analysis of fasting serum lipid profile revealed a normal high-density lipoprotein level of 1.4 mM (normal level 1.0‒1.6 mM), elevated triacylglycerides at 2.7 mM (normal limit < 2.2 mM), total cholesterol at 7.5 mM (normal limit < 6.2 mM) and low-density lipoprotein level was on upper limit at 4.9 mM (normal limit < 4.9 mM).

The endocrine evaluation showed total testosterone at 5.7 ng/mL (3.0-10.0 ng/mL), follicle-stimulating hormone at 13.2 U/L (2.0-15.0 U/L) and luteinizing hormone at 6.5 U/L (0.4-15.0 U/L) within reference values, suggesting that testosterone supplementation is efficient in this patient. Plasma levels of protein C at 122.5 U/dL (70.0-140 U/dL), protein S at 101.4% (67.5-139%), D-dimer at 64.8 ng/mL (<250.0 ng/mL), antithrombin III at 98% (80-120%) and fibrinogen at 340 mg/dL (200-375 mg/dL), as well as the serum levels of coagulation factors VII of 118% (70-140%), VIII of 115% (50-175%), IX of 110% (70-120%), XI of 101% (70-120%) and XII of 115% (60-140%) were all within the normal range. The level of serum homocysteine was 17.3 μM (5.5-16.2 μM), and the C-reactive protein level was normal at 0.9 mg/L (<5 mg/L). These results indicate a low risk for deep vein thrombosis in our patient.

The chromosomal analysis confirmed KS diagnosis with a non-mosaic 47,XXY karyotype. The patient has heterozygous 4G/5G PAI-1 genotype with normal plasma PAI-1 concentration of 30 ng/mL (3-72 ng/mL). Genetic mutations for factor II (G20210A), factor V Leiden (R506Q) and MTHFR (C677T) genes were not detected. Levels of tested antibodies were all within reference ranges, including lupus anticoagulant at 2.1 U/mL (<25 U/mL), anticardiolipin M at <2.0 U/mL (<13 U/mL) and G at 4.7 U/mL (<20.0 U/mL), antiphospholipid M at 1.3 U/mL (<15 U/mL) and G at 1.0 U/mL (<15 U/mL), and finally β2-glycoprotein I antibodies M at 1.7 U/mL (<5 U/mL), G at 1.0 U/mL (<5 U/mL) and A at 3.0 U/mL (<5 U/mL).

Taken together, we concluded that the blood analysis of biochemical and immunological parameters suggests that the patient's leg ulcer was not related to deep vein thrombosis and metabolic syndrome.

## Discussion

In this report, the high-dose acetylsalicylic acid oral intake was associated with the (re)appearance of leg ulcers in the KS patient. There is only one study so far in which acetylsalicylic acid treatment was associated with leg ulceration in KS, where, surprisingly a reduction in the size of leg ulcer was achieved with a low-dose therapy (0.3 g twice a week for one month), although the initial improvement was not maintained long-term [4].

When it was first described (1942), KS was not related to leg ulceration [5]. Almost 20 years later, Ellis et al. reported a KS patient with "large patches of inflamed skin on the lower anterior aspects of his shins which tend to ulcerate" [6]. In 1978, Howell described six KS cases complicated by hypostatic leg ulcers and discussed similar cases with arterial and venous ulceration previously reported by other authors [7]. Hypostatic leg ulceration was also described by Campbell et al. in 1980 and Verp et al. in 1983, but no underlying mechanism was proposed [8,9].

Leg ulceration in patients with KS is typically attributed to hypostasis, deep vein thrombosis and venous insufficiency as well as to platelet hyperaggregability. Such a relation was first described in a study published by Norris et al. in 1987 who emphasized the significance of supplemental androgen therapy in the context of ulcers improvement [10]. Seven years later, Veraart et al. reported two cases of KS complicated by leg ulcers, which had elevated levels of PAI-1. An inverse relationship between testosterone and PAI-1 levels was demonstrated and related to leg ulceration [11]. The significantly higher activity of PAI-1 in the group of KS patients that had leg ulcers as a complication was reported by Zollner et al. in 1997. Based on this result, authors concluded that PAI-1 is closely involved in the pathogenesis of leg ulcers in patients with KS and that therapeutic approaches aiming at PAI-1 level normalization might be helpful [12]. In our KS patient, testosterone and PAI-1 plasma levels were both within the reference interval suggesting that ulceration did not occur as a consequence of inadequate testosterone therapy and/or perturbed PAI-1 levels.

In the study of Depaire-Duclos et al. in 1997, a KS patient was found to have heterozygous factor V Leiden (R506Q) mutation and, contrary to other cases described above, supplemental androgen therapy did not show a beneficial effect on his leg ulcer [13]. The first case report on the increased activity of coagulation factor VIII-associated with venous ulcers in a patient with KS was described by Dissemond et al. in 2005, but underlying molecular defects and pathological mechanisms leading to venous thrombosis in patients with elevated coagulation factor VIII are still unclear [14]. In this case report, no high-risk gene mutations related to deep vein thrombosis were detected and the coagulation profile of the KS patient exhibited normal values.

Goto et al. suggested that formation of the leg ulcers in KS patients is attributed not only to the abnormalities of PAI-1 activity and platelet hyperaggregability but also to immunological defects due to androgen deficiency [15]. They described two KS patients with positive lupus anticoagulant and normal PAI-1 levels who had leg ulcers. In these two cases, androgen therapy gave expected results regarding leg ulcers improvement [15]. Our findings demonstrate that KS patient in our report is, and has been, a subject of successful testosterone-supplementation therapy. In addition, a normal titer of anticoagulant antibodies was detected in this patient.

Based on the literature data, it can be concluded that KS patients have an increased risk of thromboembolic events with a high prevalence of recurrent venous ulcers, vein insufficiency, and both recurrent venous and artery thromboembolism with a higher risk of deep venous thrombosis or pulmonary embolism, compared to general population. According to the recent data analysis (from 2016), there is no clear evidence of the impact of hormone replacement therapy on the risk of venous thromboembolism in patients with KS [16].

The proper concentration of gaseous nitric oxide at the location of wounded tissue seems to be essential for ulcer healing [17]. Nitric oxide is a potent cellular signaling mediator and has been reported to exert both beneficial and pathological effects, depending on the delivered dose and activation/inhibition of the nitric oxide-dependent cellular signaling pathways. For instance, insufficient production of nitric oxide is a major contributor to the dysregulation of wound healing in diabetic foot ulcer [17]. Recent reports suggest it is plausible to assume that there is a dose-dependent effect of acetylsalicylic acid on testosterone level which per se is a regulator of nitric oxide production, signaling, and turnover [18,19].

The exact biochemical pathways and mechanisms for the complex interplay between aspirin, testosterone and nitric oxide, both generally and in this particular condition (KS), have not been thoroughly elucidated. The same can be said for the systemic effects of individual substances. For instance, the antihypertensive effects of aspirin are currently a widely debated clinical issue. Although it has been suggested that aspirin lowers blood pressure and could be used for preventing hypertension, the latest guideline indicates that low-dose aspirin (75-100 mg daily) should be used infrequently in this regard, because of lack of net benefit [20].

## Conclusions

Based on the acquisition and analysis of clinical parameters, genetic profiling, and corresponding available literature evidence, the authors propose that a sudden and short-lived increase in nitric oxide concentration after administration of high doses of acetylsalicylic acid may create conditions for the appearance of leg ulcers in KS patients on a lifetime testosterone supplementation therapy. Further research on dose-dependent molecular cross-talk between testosterone, acetylsalicylic acid, and nitric oxide and its redox species, as well as the pharmacogenetic approach, are currently underway in our laboratories.
